# How long-term air pollution and its metal constituents affect type 2 diabetes mellitus prevalence? Results from Wuhan Chronic Disease Cohort

**DOI:** 10.1016/j.envres.2022.113158

**Published:** 2022-09

**Authors:** Meijin Chen, Qiujun Qin, Feifei Liu, Yixuan Wang, Chuangxin Wu, Yaqiong Yan, Hao Xiang

**Affiliations:** a Department of Global Health, School of Public Health, Wuhan University, 115# Donghu Road, Wuhan, 430071, China; b Global Health Institute, Wuhan University, 115# Donghu Road, Wuhan, 430071, China; c Wuhan Centers for Disease Control and Prevention, 288#Machang Road, Wuhan, China

**Keywords:** Type 2 diabetes, Air pollution, Metal constituents, Prevalence

## Abstract

**Background:**

Epidemiological evidence linking type 2 diabetes mellitus (T2DM) with air pollution is discrepant and most are restricted to the influences of air-pollutant mass concentration. This study aims to explore the effects of long-term exposure to air pollution and its metal constituents on T2DM prevalence in China.

**Methods:**

We used data on 10,253 adult residents from the baseline survey of Wuhan Chronic Disease Cohort in 2019. Ambient PM_2.5_, PM_10_ and NO_2_ exposure were estimated at residences based on Chinese Air Quality Reanalysis Dataset. Concentrations of 10 metal constituents were measured by 976 PM_2.5_ filter samples collected from four monitoring stations. Logistic regression models were employed to examine associations of T2DM prevalence with 3-year mean concentrations of each air pollutant and PM_2.5_ metal constituents prior to the baseline investigation.

**Results:**

A total of 673 T2DM cases (6.6%) were identified. The 3-year mean exposures to PM_2.5_, PM_10_ and NO_2_ were 50.89 μg/m^3^, 82.86 μg/m^3^, and 39.79 μg/m^3^, respectively. And interquartile range (IQR) of 10 metals in PM_2.5_ varied from 0.03 ng/m^3^ to 78.30 ng/m^3^. For 1 μg/m^3^ increment in PM_2.5_, PM_10_ and NO_2_, the odds of T2DM increased by 7.2% (95%CI: 1.026, 1.136), 3.1% (95%CI: 1.013, 1.050), and 2.1% (95%CI: 1.005, 1.038) after adjusting for potential confounders. Cd and Sb in PM_2.5_ were significant risk factors to T2DM with odds ratios of 1.350 (95%CI: 1.089, 1.673) and 1.389 (95%CI: 1.164, 1.658) for per IQR increase, respectively. Stratification analyses indicated that males and those aged ≥45 years were more susceptive to long-term air pollution.

**Conclusions:**

Long-term exposure to PM_2.5_, PM_10_ and NO_2_ increased T2DM prevalence in a Wuhan population, especially for men and middle-aged and elderly people. Moreover, T2DM was significantly associated with Cd and Sb in PM_2.5_. Further research to validate these results and to clarify the underlying mechanisms is warranted.

## Highlights

• Long-term exposure to PM2.5, PM10, and NO2 was positively associated with T2DM prevalence.
•Cd and Sb in PM2.5 were risk factors to T2DM.
•Association of air pollution and T2DM was stronger in males than in females.
•Participants aged ≥45 years old were more susceptible to air pollution exposure.

## 1 Introduction

Over the nearly three decades, type 2 diabetes mellitus (T2DM) has been a severe global epidemic with 437.9 million (5.89%) diagnosed cases in 2019 worldwide (Zheng et al., 2018). As a chronic metabolic disease, T2DM can detrimentally affect human health and quality of life with multiple complications (Braffett et al., 2020; Ng et al., 2021; Strain and Paldánius, 2018; Tsimihodimos et al., 2018; World Health Organization, 2016; Zare Sakhvidi et al., 2020). The disability-adjusted life-years (DALYs) of T2DM was 66.29 million, 2.6 times that of 1990, and around 1.47 million deaths per year can be attributed to T2DM directly (GBD, 2020). Furthermore, T2DM prevalence are expected to increase by approximately 60% by 2045, the worst in emerging economies such as China, India, and Brazil (International Diabetes Federation, 2021.; Perreault et al., 2021; Unnikrishnan et al., 2017).

In addition to genetic predisposition and lifestyle factors, air pollution is increasingly recognized as an important environmental risk to T2DM. It was reported that roughly 99% of the world's people is exposed to air pollution beyond the WHO limits, contributing to 3.2 million diabetes in 2016 (Bowe et al., 2018; World Health Organization, 2021.). And there was an alarming 108.98 per 100,000 disability-adjusted life year rate of T2DM can be attributed to PM_2.5_ (particles of ≤2.5 μm in diameter) (Liu et al., 2021). However, when emerging evidence highlights how continuous air pollution exposure contributes to the prevalence of T2DM (Eze et al., 2014; Honda et al., 2017; Lim and Thurston, 2019; Renzi et al., 2018), some other studies found inconsistent results (O'Donovan et al., 2017; Park et al., 2015; Riant et al., 2018; Strak et al., 2017). For example, evidence from a Canadian cohort study showed that for 10 μg/m^3^ elevated in PM_2.5_ exposure, there was a 28% higher prevalence odds of diabetes mellitus among female population (To et al., 2015), whereas a Netherlands study reported non-significant effect of nitrogen dioxide (NO_2_) on T2DM (OR = 0.80, 95%CI: 0.63, 1.02) (Dijkema et al., 2011), and in a nine-years American cohort study, no strong evidence supported the links between air pollution and diabetes prevalence (for PM_2.5_, the adjusted ORs was 1.16 with 95%CI: 0.94, 1.42; and for NO_2_, the adjusted ORs was 1.29 with 95%CI: 0.94, 1.76) (Park et al., 2015). To our knowledge, various chemical species of air pollutants might be one of the contributors to these inconsistencies (Evans et al., 2002; Rajagopalan and Brook, 2012; Wolf et al., 2016). From a study in 26 American communities, 1–2% higher rates of diabetes mellitus admissions were associated with per IQR increase of SO_4_^2−^ and Arsenic (As) in PM_2.5_, while organic carbon (OC) had an opposite modification (Zanobetti et al., 2009). It seems that chemical components of air pollutant mixture may considerably modify the association of air pollution and T2DM, yet receiving little attention from epidemiological research so far (Li et al., 2019b; Sun et al., 2016). Furtherly, most studies mentioned above were implemented in developed areas rather than low- and middle-income countries, where suffer the highest air pollution exposures and T2DM growth (World Health Organization, 2021).

Therefore, this research aims to provide further insights into the influence that long-term air pollution exerts on prevalent T2DM in developing countries by evaluating not simply the mass of air pollutants (PM_2.5_, PM_10_, and NO_2_), but also 10 metals in PM_2.5_, using baseline data of Wuhan Chronic Disease Cohort conducted in the largest industrial city in Central China.

## 2 Material and methods

### 2.1 Study population

We included participants from the baseline survey of Wuhan Chronic Disease Cohort, which was conducted from July 2019 to September 2019 by School of Health Sciences, Wuhan University and the Wuhan Center for Disease Control and Prevention (Wuhan CDC). Our study sites covered 13 administration districts and a national high-tech zone in Wuhan City. Regarding participants’ selection, a stratified and four-staged cluster random sampling method was applied. In the first stage, we selected three streets or townships from each district, and one or two streets from the national high-tech zone. And then, two communities were randomly chosen from each street or township by systematic sampling method. The tertiary sampling unit was household and we defined 60 households as a group. In stage 4, no less than 50 families were selected from one group and adult members (≥18 years old) in these families were included in this survey. Finally, we conducted individual questionnaires and physical measurements to 10,473 participants in the baseline survey. Further exclusion criteria included living in selected districts less than 3 years, planning to emigrate within 1 year, pregnancy, and invalid questionnaires. Thus, we excluded 220 records and 10,253 participants were remained as a sample.

### 2.2 Diagnosis of T2DM

According to the American Diabetes Association, T2DM was defined when (1) fasting blood glucose≥7.0 mmol/L; (2) and/or HbA1c ≥ 6.5%; (3) or self-reported (American Diabetes Association, 2021). In this study, we made a definite diagnosis of T2DM with self-reported status. Participants were considered as a T2DM patient when they answered “Yes” to the question “Have you been diagnosed with type 2 diabetes by doctors from a township health center or community health service center, or above?”

### 2.3 Air pollution assessment

This study measured daily, monthly, and annual average concentrations of PM_2.5_ (particles of ≤2.5 μm in diameter), PM_10_ (particles of ≤10 μm in diameter) and NO_2_ (nitrogen dioxide) of all selected streets and townships in Wuhan using the Chinese air quality reanalysis dataset (CAQRA), which was produced by the Institute of Atmospheric Physics, Chinese Academy of Sciences. CAQRA provided concentrations of PM_2.5_, PM_10_, SO_2_, NO_2_, CO, and O_3_ with a 1-h temporal frequency and a spatial resolution of 15km × 15 km in China from 2013 to 2018. In this assimilation dataset, the surface observations were assimilated with ensemble Kalman filter (EnKF) and Nested Air Quality Prediction Modeling System (NAQPMS) based on data obtained from China National Environmental Monitoring Centre (CNEMC), covering more than 1000 surface air quality monitoring sites in China. Besides, using WRF model, chemical data assimilation system (ChemDAS) further simulated wind speed (u, v), relative humidity, pressure and temperature of the surface fields. Corroborated by a 5-fold cross-validation method, the accuracy, precision, and space resolution of CAQRA were proven. (Kong et al., 2021).

Three-year mean concentrations of PM_2.5_, PM_10_ and NO_2_ (2016–2018) were exploited to assess target population's ambient long-term air pollution exposure. Residential addresses of participants at a 6-digit zip code level (street level) were converted into latitude and longitude by a geocoding tool called “map location” (https://maplocation.sjfkai.com). And then we matched PM_2.5_, PM_10_ and NO_2_ concentrations with different participants by their coordinates.

### 2.4 Determination of metals

**Fig. 1:**
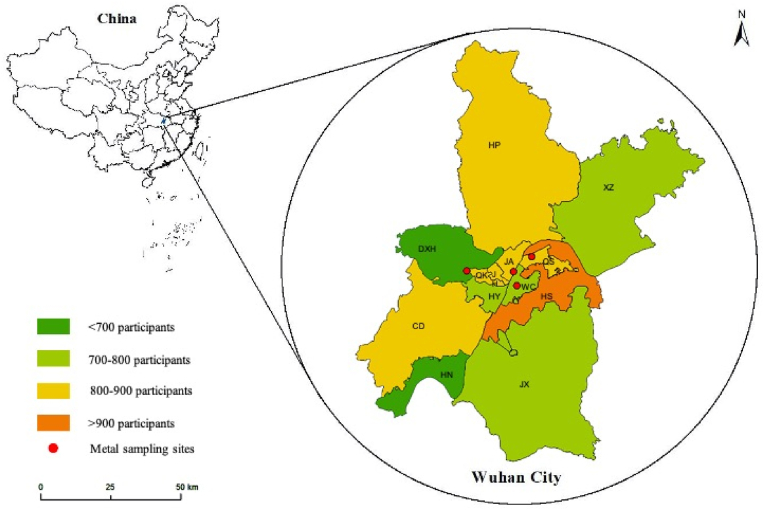
Distribution of participants and locations of four metal sampling sites in Wuhan City. Abbreviations: HP: Huangpi District; XZ: Xinzhou District; DXH: Dongxihu District; QK: Qiaokou District; JH: Jianghan District; JA: Jiangan District; QS: Qingshan District; HY: Hanyang District; WC: Wuchang District; HS: Hongshan District (the national high-tech zone is governed by Hongshan District); CD: Caidian District; HN: Hannan District; JX: Jiangxia District.

To further explore the role of exposure to chemical constituents in air pollutants, we also estimated three-year mean concentrations of Aluminum (Al), Chromium (Cr), Manganese (Mn), Nickel (Ni), Arsenic (As), Selenium (Se), Cadmium (Cd), Antimony (Sb), Thallium (Tl), and Lead (Pb) of PM_2.5_ in Wuhan City from 2016 to 2018. Using channel samplers and quartz-fiber filters with 2.5 μm (fine) fraction, 976 samples were collected from four monitoring sites located in Wuchang District (WC), Qingshan District (QS), Jiangan District (JA), and Dongxihu District (DXH) (Fig. 1) for 7 sequential days (conducted daily between 10:30 a.m. and 08:30 a.m., 22 h) each month. After being brought back to the laboratory, all filters were cut with ceramic scissors to get 1/4 effective parts for ultrasonic extraction (at 70 °C with 10 mL of 5% nitric acid for 4 h). When it cooled down to room temperature, we transferred the extraction solution to 10 mL centrifuge tubes and analyzed the metal elements with Inductively coupled plasma mass spectrometry (ICP-MS). Chemical data of PM_2.5_ was assigned with participants in terms of the nearest sampling site to their residential addresses. And we also calculated the interquartile range (IQR) of each metal element for further analysis. More information about metal sampling can be found in the supplementary material.

### 2.5 Covariates

In consideration of potential confounding factors, we performed health-related data collection with individual questionnaires. This questionnaire consisted of two parts. One was about individual containing sociodemographic characteristics, behavior patterns, body mass index (BMI), medical factors (chronic disease status and medication use), and the other part was about family characteristics such as annual household income, diet habits, and family history of T2DM. Our study assessed gender (“Male” or “Female”), age (”＜45 years old” or “≥45 years old”), marital status (“Unmarried”, “Married/Cohabiting”, “Widowed” and “Divorced/Separated”), education level (“Primary or less, ≤5 years”, “Secondary, 6–9 years” and “Tertiary, ≥10 years”), occupation (“Government employee”, “Company staff”, “Retiree”, and “Others”), annual household income (”<40,000 RMB”, “40,000–120,000 RMB”, “≥120,000RMB”), smoking (“Current”, “Ever” or “Never”), alcohol drinking (“Non-drinker” or “Drinker”), BMI, diet habits (“Vegetable intake”, “Fruits intake” and “Meat intake”, measured by intake frequency and quantity of each time over the past 12 months), the family history of T2DM (“Yes” or “No” to the question “Do your mother/father, grandmother/grandfather have T2DM?“), and the medical factors including chronic disease status (“Yes” or “No” to the question “Do you have following chronic diseases?“) and medication use (answers to the question “Do you take the medicine for hypertension?” and “Do you take the medicine for T2DM?“)

### 2.6 Statistical analysis

This study utilized logistic regression models to examine associations between PM_2.5_ (and its metal constituents), PM_10_, NO_2_, and T2DM prevalence. As for single pollutant, we estimated the odds ratios (ORs) and their 95% confidence intervals (95%CIs) of T2DM prevalence for 1 μg/m³ increment in three-year mean PM_2.5_, PM_10_, and NO_2_ concentrations, respectively. To explore the possible impact of metal constituents of PM_2.5_, the ORs for per IQR increment in metal concentrations of PM_2.5_ (including Al, Cr, Mn, Ni, As, Se, Cd, Sb, Tl, and Pb) were calculated with 95% CIs.

In each case, four adjusted models were developed to exclude an extensive set of potential confounders. Firstly, Model 1 is adjusted for sociodemographic factors including gender, age, education level, marital status, occupation, and annual family income. Secondly, Model 2 further included BMI and diet habits (intake of vegetables, fruit, and meat) as adjustments. And Model 3 was with additional adjustments for the family history of T2DM. Finally, Model 4 was a full model with the medical factors (chronic disease status and medication use) ulteriorly taken into account.

Additionally, we adopted stratification analyses to explore the heterogeneity. Stratified by gender (male/female), age (<45 years old/≥45 years old), education level (low-middle education: formal schooling time ≤9 years; high education: formal schooling time >9 years), annual family income (low-middle income: <12000RMB; high income: ≥12000RMB), and the family history of type 2 diabetes (Yes/No), potential effect modifiers were measured for the associations between PM_2.5_, PM_10_, NO_2_, and T2DM prevalence. Similarly, we defined the same stratification variables for metal concentrations analysis.

Subsequently, a series of sensitivity analyses were carried out to confirm our results. Using two-year average concentrations, we repeated the analyses mentioned above for testing models of 1 μg/m^3^ increment in single air pollutant (PM_2.5_, PM_10_, and NO_2_) concentration and per additional IQR increment in PM_2.5_ metal concentration. And for each metal constituent, the mass concentration of PM_2.5_, PM_10_, and NO_2_ were further included in the main analyses.

All statistical analyses in this study were two-sided with a significance level of p-value less than 0.05, performing in R 4.0.2.

## 3 Results

### 3.1 Population data

As summarized in Table 1, this cross-sectional study aggregately included 10,253 residents, of which 53.86% (N = 5522) were women and 41.84% (N = 4290) were less than 45 years old. Most participants were married (83.23%) and received a formal education of more than 6 years (82.46%). Over half of the cases (65.57%) reported an annual family income of more than 40,000 RMB, with an average BMI of 24.31 ± 3.70 kg/m^2^, and only 8.6% of participants have T2DM family history. In line with our definition of T2DM, there were 673 T2DM patients in this study, representing 6.6% of self-report prevalence. Among all the T2DM patients, 65.53% (N = 441) lived in central districts, close to three metal sampling sites (WC, JA, and QS).

**Table 1:** Baseline characteristics of the subjects.

Characteristics	Total	Central districts (n = 5929)	Distant urban areas (n = 4324)
**Gender**			
Male	4731 (46.14)	2742 (46.25)	1989 (46.00)
Female	5522 (53.86)	3187 (53.75)	2335 (54.00)
**Age group**			
18–24	844 (8.23)	527 (8.89)	317 (7.33)
25–44	3446 (33.61)	2024 (34.14)	1422 (32.89)
45–64	4487 (43.76)	2494 (42.06)	1993 (46.09)
65∼	1476 (14.40)	884 (14.91)	592 (13.69)
**Marital status**			
Unmarried	1272 (12.41)	837 (14.12)	435 (10.06)
Married/Cohabiting	8534 (83.23)	4826 (81.40)	3708 (85.75)
Widowed	286 (2.79)	148 (2.50)	138 (3.19)
Divorced/separated	161 (1.57)	118 (1.99)	43 (0.99)
**Education level**			
Primary or less	1799 (17.54)	490 (8.26)	1309 (30.27)
Secondary	5250 (51.21)	2960 (49.93)	2290 (52.96)
Tertiary	3204 (31.25)	2479 (41.81)	725 (16.77)
**Annual family income**^a^			
<40000	2405 (23.5)	1049 (17.70)	1356 (31.36)
40000–120000	4491 (43.80)	2493 (92.65)	1998 (46.21)
≥120000	2232 (21.77)	1561 (26.32)	671 (15.52)
**Drinking status**			
Non-drinker	7883 (76.88)	4486 (75.66)	3397 (78.56)
Drinker	2370 (23.12)	1443 (24.34)	927 (21.44)
**Smoking status**			
Current	2301 (22.44)	1283 (21.63)	1018 (23.54)
Ever	441 (4.30)	251 (4.23)	190 (4.39)
Never	7511 (73.26)	4395 (74.12)	3116 (72.04)
**Chronic disease status**			
Yes	4506 (43.95)	2539 (42.82)	2357 (54.51)
No	5747 (56.05)	3390 (57.18)	1967 (45.50)
**Medication use**^b^			
Yes	1831 (17.99)	1039 (17.52)	792 (18.32)
No	8349 (82.01)	4890 (82.48)	3459 (80.00)
**BMI**^c^	24.31 ± 3.70	23.75 ± 3.70	24.48 ± 3.64
**Vegetable intake**^d^			
<500g	9001 (87.79)	5138 (50.11)	3863 (89.33)
≥500g	1051 (10.25)	675 (11.38)	376 (8.70)
**Fruits intake**^e^			
<400g	8622 (84.09)	5035 (84.92)	3587 (82.96)
≥400g	625 (6.10)	419 (7.07)	206 (4.76)
**Meat intake**^f^			
<70g	3255 (31.75)	1835 (30.95)	1420 (32.84)
≥70g	6045 (58.96)	3622 (61.09)	2423 (56.04)
**Family history of T2DM**			
No	6993 (68.2)	3833 (64.6)	3160 (73.1)
Yes	888 (8.7)	633 (10.1)	255 (5.9)
**Prevalence of T2DM**	673 (6.6)	441 (7.4)	232 (5.4)

Central districts include Wuchang District, Qingshan District, Jiangan District, Jianghan District, Hanyang District, Hongshan District, Qiaokou District, and Donghugaoxin National High-tech Zone. Distant urban areas include Dongxihu District, Xinzhou District, Hannan District, Jiangxia District, Caidian District, Huangpi District.

a Missing data 1125.

b Missing data 73.

c Missing data 3818.

d Missing data 201.

e Missing data 1006.

f Missing data 953.

### 3.2 Air pollution and metal concentrations

As shown in Table 2, three-year average concentrations of PM_2.5_, PM_10_, and NO_2_ were 50.89 ± 2.89 μg/m^3^, 82.86 ± 8.34 μg/m^3^, 39.79 ± 9.85 μg/m^3^, respectively. Spearman rank correlation coefficients revealed that PM_2.5_ was highly correlated with PM_10_ (Spearman r = 0.93, *P* < 0.01) and moderately correlated with NO_2_ (Spearman r = 0.66, *P* < 0.01) (Table S1).

**Table 2:** Three-year average concentrations of air pollutants (μg/m^3^).

Pollutants	Mean ± SD	Max	Min	Median	IQR
PM_2.5_	50.89 ± 2.89	55.82	45.70	51.24	2.41
PM_10_	82.86 ± 8.34	96.04	68.57	83.71	9.64
NO_2_	39.79 ± 9.85	48.74	21.08	44.58	9.85

Abbreviations: PM_2.5_: particle with aerodynamic diameter ≤2.5 μm; PM_10_: particle with aerodynamic diameter ≤10 μm; NO_2_: nitrogen dioxide.

As for metal constituents of PM_2.5_ (Table 3), we can see that the three-year mean IQR of Al, Cr, Mn, and Pb were 78.30 ng/m^3^, 2.78 ng/m^3^, 2.82 ng/m^3^, and 11.9 ng/m^3^, respectively, while the other metals had an IQR lower than 1 ng/m^3^.

**Table 3:** Three-year average concentrations of metals in PM_2.5_ (ng/m^3^).

Metals	Mean ± SD	IQR	Max	Min
Al	231.756 ± 58.300	78.303	314.679	159.039
Cr	7.844 ± 3.523	2.782	13.897	5.292
Mn	27.935 ± 4.178	2.823	34.845	23.603
Ni	2.030 ± 0.405	0.276	2.728	1.726
As	6.182 ± 0.561	0.784	6.805	5.350
Se	2.794 ± 0.336	0.337	3.226	2.290
Cd	1.256 ± 0.149	0.275	1.420	1.084
Sb	2.064 ± 0.294	0.352	2.466	1.658
Tl	0.400 ± 0.032	0.028	0.453	0.367
Pb	35.327 ± 19.174	11.900	48.081	2.167

Abbreviations: PM_2.5_: particle with aerodynamic diameter ≤2.5 μm; Al: Aluminum; Cr: Chromium; Mn: Manganese; Ni: Nickel; As: Arsenic; Se: Selenium; Cd: Cadmium; Sb: Antimony; Tl: Thallium; Pb: Lead.

### 3.3 Association of air pollution with T2DM

Further analysis revealed the prevalence of T2DM had significantly positive correlations with long-term exposure to PM_2.5_, PM_10_, and NO_2_ (Table 4). In Model 4, per 1 μg/m^3^ elevated in three-year mean concentrations of PM_2.5_ raised odds of T2DM by 7.2% (95%CI:1.026, 1.136). In terms of PM_10_ and NO_2_, relatively weaker positive results were observed that every 1 μg/m^3^ increment only predicted 3.1% (95%CI:1.013, 1.050) and 2.1% (95%CI:1.005, 1.038) higher odds of T2DM, respectively.

**Table 4:** Model results as odds ratios with 95% confidence interval for the associations between T2DM prevalence and three-year average concentrations of each air pollutant.

	Crude Model	Model 1	Model 2	Model 3	Model 4
PM_2.5_	1.045 (1.018 1.073)	1.074 (1.035 1.114)	1.095 (1.049 1.144)	1.075 (1.021 1.132)	1.072 (1.026 1.136)
PM_10_	1.021 (1.012 1.031)	1.031 (1.018 1.044)	1.037 (1.021 1.053)	1.030 (1.012 1.049)	1.031 (1.013 1.050)
NO_2_	1.021 (1.012 1.029)	1.019 (1.007 1.031)	1.024 (1.010 1.039)	1.019 (1.002 1.036)	1.021 (1.005 1.038)

Model 1: adjusted for gender, age, annual family income, education level, marital status, and occupation.

Model 2: adjusted for gender, age, annual family income, education level, marital status, occupation, BMI, and diet habits (vegetable intake, meat intake, and fruits intake).

Model 3: adjusted for gender, age, annual family income, education level, marital status, occupation, BMI, diet habits (vegetable intake, meat intake, and fruits intake), and family history of T2DM.

Model 4: adjusted for gender, age, annual family income, education level, marital status, occupation, BMI, diet habits (vegetable intake, meat intake, and fruits intake), family history of T2DM, and medical factors (chronic disease status and medication use).

Abbreviations: T2DM: type 2 diabetes mellitus; PM_2.5_: particle with aerodynamic diameter ≤2.5 μm; PM_10_: particle with aerodynamic diameter ≤10 μm; NO_2_: nitrogen dioxide.

**Fig. 2:**
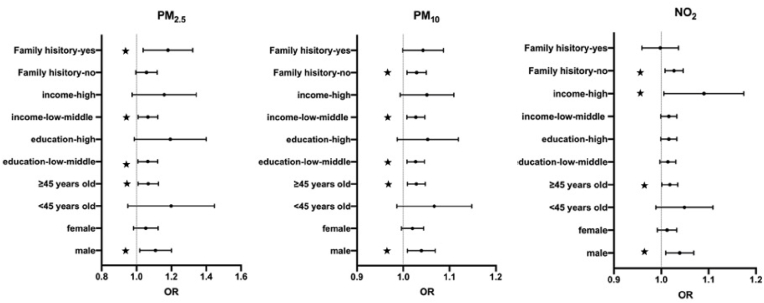
Stratified analysis of PM_2.5_, PM_10_, and NO_2._ Low-middle income: annual family income <120,000 RMB; high income: annual family income ≥120,000 RMB; low-middle education: formal schooling time ≤9 years; high education: formal schooling time >9 years.  represents significant interaction (*P* < 0.05). Abbreviations: PM_2.5_: particle with aerodynamic diameter ≤2.5 μm; PM_10_: particle with aerodynamic diameter ≤10 μm; NO_2_: nitrogen dioxide.

The results of stratification analyses are illustrated in Fig. 2. As expected, gender (male) and age (≥45 years old) had significant interaction effects on the associations between PM_2.5_, PM_10_, NO_2_ and T2DM prevalence. From Table S2, males were more vulnerable to long-term exposure to PM_2.5_ (OR = 1.107, 95%CI: 1.019, 1.201), PM_10_ (OR = 1.039, 95%CI: 1.009, 1.069), and NO_2_ (OR = 1.038, 95%CI: 1.010, 1.069) when compared to females. And in subgroup analysis of age, PM_2.5_, PM_10_, and NO_2_ exposure's impacts on people ≥45 years old were statistically significant with the increase in odds of T2DM by 6.6% (95%CI: 1.009, 1.126), 2.8% (95%CI: 1.009, 1.048), and 1.8% (95%CI: 1.001, 1.035), respectively. It stands out in Fig. 2 that the effect of education level in NO_2_ differed from PM_2.5_ and PM_10_. Compared to participants with high education level, those who received low-middle education faced a pronounced risk of T2DM when exposed to PM_2.5_ (OR = 1.063, 95%CI: 1.008, 1.122) and PM_10_ (OR = 1.027, 95%CI: 1.008, 1.046), while no significant influence of education was detected in NO_2_. And the high level of annual family income also played a significant modified role in association of NO_2_ and T2DM, increasing the odds of T2DM by 8.2% (95%CI:1.010, 1.178). By contrast, people with low-middle annual family income were vulnerable to PM_2.5_ (OR = 1.065, 95%CI: 1.009, 1.122) and PM_10_ (OR = 1.027, 95%CI: 1.008, 1.047) significantly.

### 3.4 Associations between metal concentrations and T2DM

**Fig. 3:**
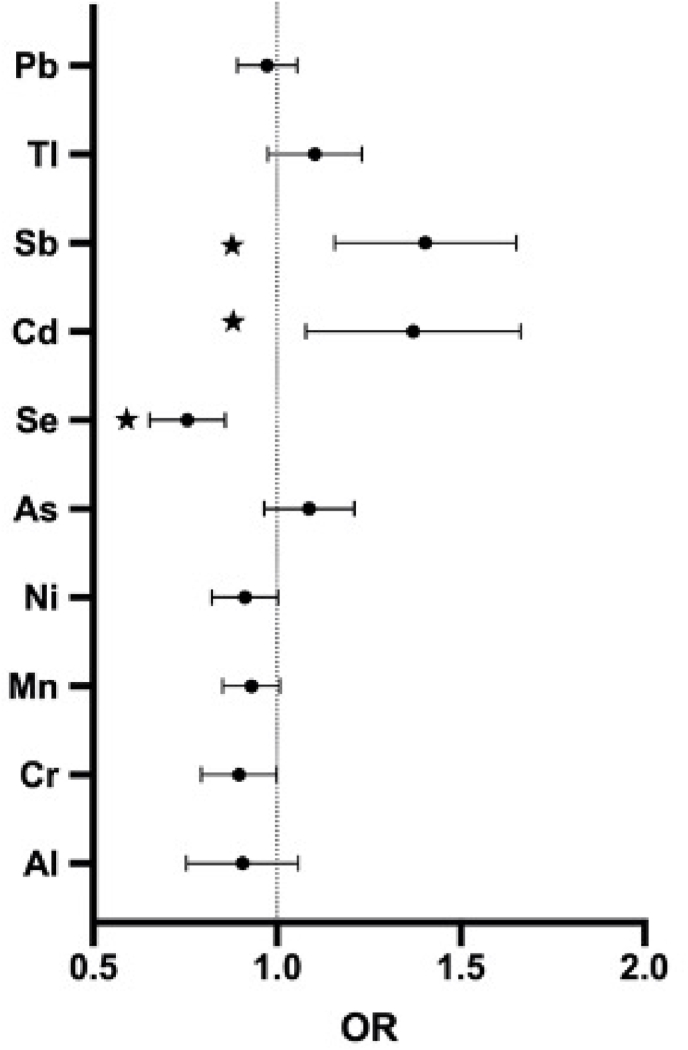
Results of adjusted model of metal constituents in PM_2.5._ This model adjusted for gender, age, annual family income, education level, marital status, occupation, BMI, diet habits (vegetable intake, meat intake, and fruits intake), family history of T2DM, and medical factors (chronic disease status and medication use).  represents significant results (*P* < 0.05). Abbreviations: PM2.5: particle with aerodynamic diameter ≤2.5 μm; Al: Aluminum; Cr: Chromium; Mn: Manganese; Ni: Nickel; As: Arsenic; Se: Selenium; Cd: Cadmium; Sb: Antimony; Tl: Thallium; Pb: Lead.

Fig. 3 further demonstrates the relationship of metal concentration in PM_2.5_ and T2DM prevalence. It was proven in Table S3 that Cd and Sb were significant risk factors for T2DM with ORs of 1.350 (95%CI:1.089, 1.673) and 1.389 (95%CI:1.164, 1.658) for every additional IQR, respectively. On the contrary, Se exerted protective effects on T2DM with odds ratio of 0.751 (95%CI:0.655, 0.861). Table S4 shows the results of metal concentration stratified analysis. Similar to the results of air pollution stratification analyses, males and those aged ≥45 years had higher odds of T2DM with per IQR increment of Cd and Sb in PM_2.5_.

### 3.5 Sensitivity analysis

According to sensitivity analyses, the associations between long-term air pollution and T2DM prevalence were stable for the two-year exposure data. As shown in Table S6, odds ratios of T2DM prevalence remained statistically significant with 1 μg/m^3^ increase in PM_2.5_ (OR = 1.083, 95%CI:1.024, 1.144), PM_10_ (OR = 1.033, 95%CI: 1.013, 1.054), and NO_2_ (OR = 1.020, 95%CI:1.004, 1.037), respectively, which were consistent with those from main analyses. Besides, in the sensitivity analysis of 2-year average concentration of metal constituents in PM_2.5_, associations of Cd and Sb with T2DM prevalence were robust after controlling air-pollutant mass concentration (PM_2.5_, PM_10_, and NO_2_), strongly supporting our primary conclusions. (Table S8).

## 4 Discussion

As far as we learn, this is the first study in China considering both the concentration of ambient air pollutants and its metal constituents in the associations of long-term air pollution and T2DM prevalence. In the present research, three-year average exposures to PM_2.5_, PM_10_, and NO_2_ showed significantly positive associations with T2DM prevalence among 10,253 adult residents in Wuhan, especially for males and those aged ≥45 years old. Furthermore, Cd and Sb in PM_2.5_ were significantly related to higher prevalence of T2DM. All these associations were robust and consistent across sensitivity analyses with 2-year exposure windows.

Public attention has been rising on how long-term ambient air pollution exerts an influence on T2DM, yet epidemiological evidence for developing countries, like China, was disproportionately less. Our research suggested that the odds of T2DM prevalence was elevated by 7.2% (95%CI:1.026,1.136), 3.1% (95%CI:1.013,1.050), and 2.1% (95%CI:1.005,1.038) for additional 1 μg/m^3^ increment in PM_2.5_, PM_10_, and NO_2_, respectively, which were similar to earlier findings in rural China that with every 1 μg/m^3^ increment in PM_2.5_ and NO_2_, there are 6.8% and 5.0% higher odds of T2DM prevalence, respectively (Liu et al., 2019). Our outcomes were also in line with a recent meta-analysis, reporting ORs of 1.08, 1.10, and 1.07 in T2DM prevalence with a 10 μg/m^3^ increment in PM_2.5_, PM_10_, and NO_2_, respectively (Yang et al., 2020). By comparison, the evidence from China, where many people suffered from polluted air, showed stronger associations than those from developed countries. According to a meta-analysis focusing on North America and Europe, per 10 μg/m^3^ elevated in PM_2.5_ and NO_2_ increased 10% and 8% odds of type 2 diabetes, respectively (Eze et al., 2015). Specifically, in Italy, for an additional 10 μg/m^3^ increment in PM_2.5_, PM_10_, and NO_2_, self-reported diabetes increased by 4%, 4%, and 3%, respectively (Orioli et al., 2018). It was suggested that the degree of this relevance may vary with different levels of exposure and population characteristics (Y. Y.Li et al., 2019c). Effect values estimated in this research were greater probably because of serious air pollution in Wuhan, which exceeded the maximum limit according to the Chinese government (annual limit values for PM_2.5_, 35 μg/m^3^; for PM_10_, 70 μg/m^3^; for NO_2_, 40 μg/m^3^) (Ambient air quality standards,GB3095-2012.), susceptibility of target population, different analytical method, and sample size.

Until now, the mechanisms linking air pollution and T2DM have not been clear, and several hypotheses exist. Some researches demonstrated that exposure to PM_2.5,_ PM_10_ and NO_2_ might cause insulin resistance and reduced glucose tolerance, raising the risk of T2DM (Kelishadi et al., 2009; Rajagopalan and Brook, 2012; van der Pol et al., 2019). Also, oxidative stress is widely recognized as one of the key factors of linking air pollution and T2DM, which may generate a sequence of biological chemical events by inducing lipid peroxidation, activating pro-inflammatory factors and mediating inflammatory responses (Lim and Thurston, 2019).

Besides, our study provided some clues for the independent effect of PM_2.5_ metal constituents on T2DM. Though the pathogenic mechanism of air pollution on T2DM remains equivocal, it was suggested that toxic constituents of pollutants may play a crucial role in triggering oxidative stress and systemic inflammation, suppressing insulin signaling, and contributing to glycolipid metabolism disorder (Lucht et al., 2019; Sanjay Sanjay Rajagopalan and Brook, 2012). In this study, we identified Cd and Sb in PM_2.5_ as significant risk factors for T2DM (for an IQR increment in Cd, the odds of T2DM prevalence were increased by 35.0% with 95%CI:1.089, 1.673; and for Sb were 58.9% with 95%CI:1.164, 1.658). Because of the small particle size (≤2.5 μm), it is easy for PM_2.5_-bound toxic heavy metals (e.g., Cd and Sb) to penetrate lungs and enter blood circulation system, increasing insulin antagonism by inducing inflammation (Gangwar et al., 2020; Xie et al., 2020). Some biological evidence showed that Cd and Sb can activate pathways related to diabetes, including oxidative stress response, chronic pancreatic injury, or insulin transcription and secretion, which may interfere with the human endocrine system and lead to apoptosis of islet β cells, resulting in insulin antagonism (Rehman et al., 2018; Wang et al., 2020; Zhang et al., 2021). By contrary, Se was related to lower T2DM prevalence with odds ratio of 0.751 (95%CI:0.655, 0.861) in the main models but not significant in sensitivity analysis for 2-year average concentration (OR = 0.945; 95%CI: 0.817, 1.099). Se, an active ingredient constituting glutathione peroxidase (GSH-Px), is usually considered as a part of the antioxidant system and participates in the detoxification of peroxides through glutathione peroxidase (mainly GPx 1), thereby reducing the effect of oxidative stress on islet β cells (Mueller et al., 2009).

This is one of the few studies exploring the association between metal components in PM_2.5_ and the prevalence of T2DM. Human epidemiological researches on the associations are limited, and most of them are confined to gestational diabetes mellitus (GDM) (Rammah et al., 2020; Yu et al., 2020; Zheng et al., 2021). Specifically, a retrospective cohort study carried out in China suggested that an IQR increase in nitrate, organic matter, and black carbon was associated with 13%, 14%, and 15% higher risks of GDM (Yu et al., 2020). Another Florida study including 2,078,669 women found that exposures to NH_4_^+^ and OM were positively related to GDM, whereas mineral dust may play a protective role. Outcome disparities in these studies could be owing to differences in study sites, seasons, and particle mixture (Xie et al., 2019; Yang et al., 2019). Considering the varying effects, more research should be undertaken to investigate how specified metal constituents in PM_2.5_ impact T2DM.

Furthermore, stratified analyses by gender suggested that males in Wuhan were more susceptible to long-term air pollution, which accorded with earlier studies in China, Korea, Canada, and Europe (Emerging Risk Factors Collaboration et al., 2010; Hwang et al., 2020; Li et al., 2019a; Lipscombe and Hux, 2007; Weinmayr et al., 2015). In addition to exposure pattern, work specialization, and lifestyles, one possible explanation may be sexual differences in hormonal characteristics (Clougherty, 2010; Pomatto et al., 2017; Schirmer et al., 2016). In our study, participants aged 45 and older were more vulnerable to type 2 diabetes under long-term exposure to PM_2.5_, PM_10_, and NO_2_. Similar conclusions can be found in numerous previous studies, suggesting stronger positive associations between air pollution and T2DM among middle-aged and elderly population (Chen et al., 2016; Eze et al., 2014; Honda et al., 2017; Liu et al., 2019; Suryadhi et al., 2020). This may be owing to age-related decline of physiological function, physical reserves, immune system, stress response and so on (Collins, 1987; Sacks et al., 2011; Vasto et al., 2007). Also, the elderly are more likely to have underlying medical conditions such as respiratory disease and cardiovascular disease, which may worsen the impact of persistent air pollution on oxidative stress and vasoconstriction (Ben-Shlomo and Kuh, 2002; Strain and Paldánius, 2018). Another possible reason is that compared with the young, older people are more frequently exposed to ambient air pollution because of outdoor activity (To et al., 2015). Interestingly, our study further indicated that different levels of income earners were unequally affected by PM_2.5_, PM_10_, and NO_2_. For low-middle income earners, in close agreement with results of previous research, PM_2.5_ and PM_10_ were significant risk factors (Jbaily et al., 2020; Ruiz et al., 2017; Wu et al., 2019). However, participants who have high income were more susceptible to NO_2_ exposure. We speculated that it may be partly attributed to higher exposure of traffic-related air pollution, of which NO_2_ is usually identified as a primary marker (Anttila et al., 2011; Bechle et al., 2011; Carslaw, 2005; Kamińska, 2019). To be specific, high earners commonly live in downtown areas with heavy traffic, have a higher proportion of vehicle ownership and drive a lot, probably raising their risk from automobile exhaust emission than low-middle income ones (Danish and Madany, 1992; Stieb et al., 2016; Tonne et al., 2018).

Still, several limitations of this research should be noted. Firstly, the prevalence of T2DM was assessed by self-report data, which may bring biases. Secondly, daily information of individual activity pattern was unavailable. Though we employed a validated air pollution assessment model with high precision, it was imprecise to estimate personal exposure with a proxy rather than direct measurement (Guo et al., 2020; Hu et al., 2020). To minimize bias, we will collect more individual data in follow-up visits. Thirdly, the concentration of metal constituents were measured by samples from four monitoring sites, which would be insufficient to evaluate the effects of spatial heterogeneity. Also, this study mainly evaluated the effects of several air pollutants and metal components separately, while exposure to air pollution is complicated and simultaneous, known as “joint exposure”, which would augment these associations. Thus, this research may underestimate the health risks of air pollution (Li et al., 2021a, Li et al., 2021b). Finally, our results showed that specific metal components were related to higher T2DM prevalence, however, the detailed mechanism of how they provoke T2DM is still poorly understood. To develop a full picture of associations between air pollution and T2DM prevalence, further experimental research in consideration of personal joint exposure are necessary.

## 5 Conclusions

In general, this study indicated that long-term exposure to ambient air pollution and its metal constituents (Cd and Sb in PM_2.5_) was positively related to the increased prevalence of T2DM, especially for males and older people. These results complemented those of earlier studies in developing countries. Further research is needed not only to confirm our findings, but also to illustrate the mechanism underlying these associations.

## Funding

We thank the 10.13039/100000865Bill & Melinda Gates Foundation [Grant Number OOP1148464], Wuhan Center for Disease Control & Prevention [Grant Number 1602-250000196] and Wuhan Municipal Health Commission [Grant Number WY19A01] for funding.

## Declaration of competing interest

The authors declare that they have no known competing financial interests or personal relationships that could have appeared to influence the work reported in this paper.
