# Supplementary material for: How long-term air pollution and its metal constituents affect type 2 diabetes mellitus prevalence? Results from Wuhan Chronic Disease Cohort
Source: Environ Res. 2022 Sep;212(Pt A):113158. doi: 10.1016/j.envres.2022.113158 (PMC9227727; doi:10.1016/j.envres.2022.113158)
Supplement: Multimedia component 1 [file mmc1.docx]

**Measurements of metals in PM_2.5_ (particle with aerodynamic diameter ≤2.5 μm)**

The sampling quartz microfiber filters (90mm, Whatman International Ltd., Maidstone, England) of this study were collected from CDCs of different districts (Wuchang CDC, Qingshan CDC, Dongxihu CDC, Jiangan CDC). A total of 7 days of continuous sampling per month in every year, the samples were collected 22 hours a day (from 10:30 am to 8:30 am) using PM_2.5_ airborne particulate matter sampler (Wuhan Tianhong Environmental Protection Industry Co., TH-1000CII). After bringing back to the laboratory, the filters were cut to four pieces equally, and 1/4 filters were used to detect. The way of extraction was that at 70 ℃ with 10 mL of 5% nitric acid for 4 hours and then use ultrasonic extraction. When it cooled down to room temperature, we transferred the extraction solution to 10 mL centrifuge tubes and analyzed the metal elements with Inductively coupled plasma mass spectrometry (ThermoFisher, ICP-MS).

**Table S1** Spearman correlation coefficients (p-value) of 3-year average air pollutant concentrations

| Air pollutant | PM_10_ | PM_2.5_ | NO_2_ |
| --- | --- | --- | --- |
| PM_10_ | 1.00 | **0.93(< 0.01)** | **0.72 (<0.01)** |
| PM_2.5_ |  | 1.00 | **0.66** (**<0.01**) |
| NO_2_ |  |  | 1.00 |

Abbreviations: PM_10_, particle with aerodynamic diameter ≤10 μm; PM_2.5_, particle with aerodynamic diameter ≤2.5 μm; NO_2_, nitrogen dioxide.

**Table S2** Model results as odds ratios with 95% confidence interval for the stratified analysis of associations between air pollutants and T2DM prevalence

|  | PM_2.5_ | PM_10_ | NO_2_ |
| --- | --- | --- | --- |
| Male | **1.107(1.019 1.201)** | **1.039(1.009 1.069)** | **1.038(1.010 1.069)** |
| Female | 1.052(0.983 1.124) | 1.020(0.996 1.044) | 1.012(0.992 1.033) |
| <45 years old | 1.183(0.958 1.454) | 1.065(0.987 1.149) | 1.045(0.991 1.111) |
| ≥45 years old | **1.066(1.009 1.126)** | **1.028(1.009 1.048)** | **1.018(1.001 1.035)** |
| Low-middle income | **1.065(1.009 1.122)** | **1.027(1.008 1.047)** | **1.016(0.999 1.033)** |
| High income | 1.148(0.981 1.348) | 1.050(0.994 1.110) | **1.082(1.010 1.178)** |
| Low-middle education | **1.063(1.008 1.122)** | **1.027(1.008 1.046)** | 1.014(0.997 1.031) |
| High education | 1.181(0.994 1.406) | 1.051(0.988 1.120) | 1.016(0.999 1.033) |
| Family history of T2DM (Yes) | **1.174(1.042 1.326)** | 1.042(0.999 1.087) | 0.997(0.960 1.037) |
| Family history of T2DM (No) | 1.056(0.996 1.119) | **1.029(1.008 1.050)** | **1.027(1.008 1.046)** |

Abbreviations: T2DM: type 2 diabetes mellitus; PM_2.5_, particle with aerodynamic diameter ≤2.5 μm; PM_10_, particle with aerodynamic diameter ≤10 μm; NO_2_, nitrogen dioxide.

**Table S3** Model results as odds ratios with 95% confidence interval for the associations between T2DM prevalence and three-year average concentrations of PM_2.5_ metal constituents

|  | Crude Model | Model 1 | Model 2 | Model 3 | Model4 |
| --- | --- | --- | --- | --- | --- |
| Al | 0.964(0.874 1.063) | 0.932(0.8341.041) | 0.925(0.802 1.066) | 0.901(0.762 1.065) | 0.897(0.758 1.062) |
| Cr | 0.974(0.912 1.038) | 0.918(0.8530.986) | 0.890 (0.8040.980-) | 0.894(0.796 0.999) | 0.894(0.795 1.000) |
| Mn | 0.956(0.910 1.005) | 0.963(0.912 1.017) | 0.933 (0.8681.002) | 0.932(0.857 1.012) | 0.928(0.853 1.009) |
| Ni | 0.947(0.900 0.995) | 0.946(0.894 0.999) | 0.908 (0.8420.976) | 0.907(0.832 0.987) | 0.903(0.827 0.984) |
| As | 1.082(0.968 1.212) | 1.219(1.077 1.382) | 1.309 (1.1081.550) | 1.325(1.090 1.616) | 1.326(1.088 1.619) |
| Se | 0.866(0.799 0.939) | 0.849(0.779 0.926) | 0.746 (0.6640.838) | 0.755(0.659 0.865) | 0.751(0.655 0.861) |
| Cd | 1.206(1.039 1.398) | 1.370(1.166 1.609) | 1.382 (1.1521.659) | 1.346(1.087 1.667) | 1.350(1.089 1.673) |
| Sb | 1.143(1.032 1.267) | 1.224(1.080 1.386) | 1.409 (1.2121.639) | 1.386(1.163 1.652) | 1.389(1.164 1.658) |
| Tl | 1.008(0.942 1.077) | 1.081 (1.003 1.164) | 1.076 (0.9751.185) | 1.099(0.979 1.233) | 1.099(0.977 1.233) |
| Pb | 1.010(0.963 1.062) | 0.969(0.918 1.023) | 0.988 (0.9211.061) | 0.969(0.893 1.055) | 0.970(0.893 1.056) |

Model 1: adjusted for gender, age, annual family income, education level, marital status, and occupation

Model 2: adjusted for gender, age, annual family income, education level, marital status, occupation, BMI, and diet habits (vegetable intake, meat intake, and fruits intake)

Model 3: adjusted for gender, age, annual family income, education level, marital status, occupation, BMI, diet habits (vegetable intake, meat intake, and fruits intake), and family history of T2DM

Model 4: adjusted for gender, age, annual family income, education level, marital status, occupation, BMI, diet habits (vegetable intake, meat intake, and fruits intake), family history of T2DM, and medical factors (chronic disease status and medication use)

Abbreviations: T2DM: type 2 diabetes mellitus; PM_2.5_, particle with aerodynamic diameter ≤2.5 μm; Al: Aluminum; Cr: Chromium; Mn: Manganese; Ni: Nickel; As: Arsenic; Se: Selenium; Cd: Cadmium; Sb: Antimony; Tl: Thallium; Pb: Lead.

**Table S4** Model results as odds ratios with 95% confidence interval for the stratified analysis of PM_2.5_ metal constituents

|  | Male | Female | <45 years old | ≥45 years old | Low-middle education | High education | Low-middle income | High income | Family history of T2DM-Yes | Family history of T2DM-No |
| --- | --- | --- | --- | --- | --- | --- | --- | --- | --- | --- |
| Al | 0.892(0.6861.157) | 0.951(0.7591.190) | 1.050(0.783 1.404) | 0.909(0.733 1.127) | 0.907(0.756 1.088) | 1.067(0.851 1.335) | 0.967(0.825 1.133) | 0.793(0.503 1.209) | 0.875(0.640 1.188) | 0.961(0.791 1.168) |
| Cr | 0.878 (0.729 1.045) | 0.871 (0.747 1.008) | 0.865(0.727 1.021) | 0.868(0.736 1.013) | 0.958(0.855 1.070) | 0.910(0.780 1.053) | 0.951(0.861 1.048) | 0.810(0.577 1.082) | 0.804(0.650 0.982) | 0.970(0.871 1.075) |
| Mn | 0.901 (0.789 1.026) | 0.972(0.8691.084) | 1.011(0.869 1.171) | 0.944(0.851 1.047) | 0.950(0.869 1.036) | 1.005(0.888 1.133) | 0.976(0.903 1.053) | 0.839(0.636 1.077) | 0.847(0.705 1.008) | 0.986(0.908 1.069) |
| Ni | 0.871 (0.758 0.994) | 0.947(0.8441.059) | 0.977(0.833 1.139) | 0.922(0.828 1.023) | 0.937(0.855 1.023) | 0.986(0.869 1.113) | 0.964(0.890 1.042) | 0.775(0.563 1.013) | 0.787(0.644 0.945) | 0.900(1.063 1.023) |
| As | 1.391 (1.023 1.909) | 1.327(1.0291.718) | 1.281(0.960 1.722) | 1.398(1.067 1.839) | 1.151(0.941 1.411) | 1.137(0.894 1.453) | 1.124(0.945 1.338) | 1.568(0.983 2.621) | 1.593(1.130 2.277) | 1.081(0.8970 1.306) |
| Se | **0.690(0.5610.849)** | 0.869(0.7401.023) | 0.907(0.751 1.097) | **0.763(0.641 0.910)** | 0.912(0.810 1.029) | 0.840(0.690 1.027) | 0.891(0.788 1.010) | 0.660(0.362 1.195) | **0.575(0.439 0.748)** | 0.947(0.828 1.085) |
| Cd | **1.829(1.2342.721)** | 1.304(0.9861.720) | 1.227(0.889 1.696) | **1.689(1.203 2.366)** | 1.285(0.988 1.671) | 1.377(0.938 2.030) | 1.220(0.971 1.532) | 1.445(0.580 3.786) | **1.695(1.172 2.473)** | 1.141(0.8911.457) |
| Sb | **1.510 (1.154 1.983)** | 1.141 (0.968 1.343 | 1.146(0.931 1.411) | **1.458(1.145 1.856)** | 1.153(0.967 1.375) | 1.256(0.993 1.593) | 1.143(0.980 1.332) | 1.407(0.690 3.007) | **1.801(1.316 2.493)** | 1.080(0.915 1.273) |
| Tl | 1.105(0.9481.283) | 1.088(0.9051.305) | 1.068(0.895 1.271) | 1.124(0.957 1.315) | 1.067(0.939 1.208) | 1.005(0.874 1.154) | 1.040(0.933 1.157) | 1.160(0.904 1.494) | 1.125(0.927 1.366) | 1.038(0.923 1.165) |
| Pb | 0.992 (0.871 1.136) | 0.966 (0.867 1.082) | 0.997(0.878 1.136) | 0.959(0.857 1.080) | 0.969(0.886 1.064) | 1.026(0.929 1.137) | 0.990(0.916 1.072) | 0.937(0.785 1.119) | 0.981(0.855 1.130) | 0.981(0.903 1.070) |

Model 1: adjusted for gender, age, annual family income, education level, marital status, and occupation

Model 2: adjusted for gender, age, annual family income, education level, marital status, occupation, BMI, and diet habits (vegetable intake, meat intake, and fruits intake)

Model 3: adjusted for gender, age, annual family income, education level, marital status, occupation, BMI, diet habits (vegetable intake, meat intake, and fruits intake), and family history of T2DM

Model 4: adjusted for gender, age, annual family income, education level, marital status, occupation, BMI, diet habits (vegetable intake, meat intake, and fruits intake), family history of T2DM, and medical factors (chronic disease status and medication use)

Abbreviations: PM_2.5_, particle with aerodynamic diameter ≤2.5 μm; Al: Aluminum; Cr: Chromium; Mn: Manganese; Ni: Nickel; As: Arsenic; Se: Selenium; Cd: Cadmium; Sb: Antimony; Tl: Thallium; Pb: Lead.

**Table S5** Two-year average concentrations of air pollutants

| Pollutants | Mean±SD | Max | Min | Median | IQR |
| --- | --- | --- | --- | --- | --- |
| PM_2.5_ | 49.31±2.57 | 53.75 | 45.24 | 49.24 | 1.73 |
| PM_10_ | 80.49±7.51 | 92.47 | 68.52 | 80.74 | 7.74 |
| NO_2_ | 40.11±9.95 | 49.29 | 21.39 | 44.83 | 19.96 |

Abbreviations: PM2.5, particle with aerodynamic diameter ≤2.5 μm; PM10, particle with aerodynamic diameter ≤10 μm; NO2, nitrogen dioxide.

**Table S6** Sensitivity model results as odds ratios with 95% confidence interval for the associations between T2DM prevalence and two-year average concentrations of air pollutants

|  | Crude Model | Model 1 | Model 2 | Model 3 | Model 4 |
| --- | --- | --- | --- | --- | --- |
| PM_2.5_ | 1.039(1.009 1.070) | 1.066(1.030 1.103) | 1.103(1.052 1.156) | 1.079(1.020 1.141) | 1.083(1.024 1.144) |
| PM_10_ | 1.021(1.011 1.032) | 1.031(1.018 1.044) | 1.041(1.023 1.059) | 1.032(1.012 1.053) | 1.033(1.013 1.054) |
| NO_2_ | 1.018(1.010 1.027) | 1.022(1.012 1.033) | 1.023(1.009 1.038) | 1.018(1.002 1.035) | 1.020(1.004 1.037) |

Model 1: adjusted for gender, age, annual family income, education level, marital status, and occupation

Model 2: adjusted for gender, age, annual family income, education level, marital status, occupation, BMI, and diet habits (vegetable intake, meat intake, and fruits intake)

Model 3: adjusted for gender, age, annual family income, education level, marital status, occupation, BMI, diet habits (vegetable intake, meat intake, and fruits intake), and family history of T2DM

Model 4: adjusted for gender, age, annual family income, education level, marital status, occupation, BMI, diet habits (vegetable intake, meat intake, and fruits intake), family history of T2DM, and medical factors (chronic disease status and medication use)

Abbreviations: T2DM: type 2 diabetes mellitus; PM_2.5_, particle with aerodynamic diameter ≤2.5 μm; PM_10_, particle with aerodynamic diameter ≤10 μm; NO_2_, nitrogen dioxide.

| Metals | Mean±SD | IQR | Max | Min |
| --- | --- | --- | --- | --- |
| Al | 245.155±54.486 | 93.479 | 313.127 | 182.215 |
| Cr | 9.570±4.800 | 3.006 | 17.826 | 5.900 |
| Mn | 29.231±6.905 | 6.344 | 40.859 | 23.177 |
| Ni | 2.190±0.455 | 0.306 | 2.969 | 1.818 |
| As | 5.846±0.604 | 0.514 | 6.488 | 4.858 |
| Se | 2.654±0.685 | 0.550 | 3.431 | 1.552 |
| Cd | 1.237±0.184 | 0.302 | 1.444 | 0.992 |
| Sb | 2.072±0.310 | 0.300 | 2.466 | 1.602 |
| Tl | 0.384±0.038 | 0.046 | 0.430 | 0.327 |
| Pb | 35.365±17.933 | 16.427 | 48.140 | 2.167 |

**Table S7** Two-year average concentrations of PM_2.5_ metal constituents (ng/m^3^)

Abbreviations: PM_2.5_, particle with aerodynamic diameter ≤2.5 μm; Al: Aluminum; Cr: Chromium; Mn: Manganese; Ni: Nickel; As: Arsenic; Se: Selenium; Cd: Cadmium; Sb: Antimony; Tl: Thallium; Pb: Lead.

**Table S8** Sensitivity model results as odds ratios with 95% confidence interval for the associations between T2DM prevalence and two-year average concentrations of PM_2.5_ metal constituents

|  | 2-year average | Model 1 | Model 2 | Model 3 |
| --- | --- | --- | --- | --- |
| Al | 0.895(0.701 1.144) | 0.991(0.833 1.179) | 0.989(0.832 1,175) | 0.965(0.813 1.144) |
| Cr | 1.013(0.893 1.145) | 0.917(0.815 1.028) | 0.910(0.809 1.019) | 0.883(0.786 0.987) |
| Mn | 0.972(0.838 1.120) | 0.982(0.898 1.074) | 0.985(0.901 1.077) | 0.958(0.880 1.043) |
| Ni | 0.917(0.812 1.034) | 0.963(0.877 1.057) | 0.966(0.880 1059) | 0.933(0.853 1.018) |
| As | 1.019(0.903 1.150) | 1.202(0.976 1.483) | 1.213(0.990 1.490) | 1.296(1.066 1.579) |
| Se | 0.945(0.817 1.099) | 0.803(0.677 0.954) | 0.815(0.691 0.963) | 0.769(0.669 0.885) |
| Cd | 1.237(1.031 1.481) | 1.423(1.056 1.915) | 1.412(1.061 1.879) | 1.566(1.209 2.028) |
| Sb | 1.117(1.012 1.232) | 1.288(1.054 1.576) | 1.281(1.056 1.558) | 1.381(1.159 1.647) |
| Tl | 1.039(0.908 1.187) | 1.045(0.928 1.175) | 1.055(0.938 1.184) | 1.071(0.953 1.201) |
| Pb | 0.964(0.854 1.091) | 0.995(0.917 1.083) | 0.990(0.912 1.076) | 0.993(0.914 1.080) |

Model 1: adjusted for concentrations of PM_2.5_

Model 2: adjusted for concentrations of PM_10_

Model 3: adjusted for concentrations of NO_2_

Abbreviations: PM_2.5_, particle with aerodynamic diameter ≤2.5 μm; PM_10_, particle with aerodynamic diameter ≤10 μm; NO_2_, nitrogen dioxide; T2DM: type 2 diabetes mellitus; Al: Aluminum; Cr: Chromium; Mn: Manganese; Ni: Nickel; As: Arsenic; Se: Selenium; Cd: Cadmium; Sb: Antimony; Tl: Thallium; Pb: Lead.
